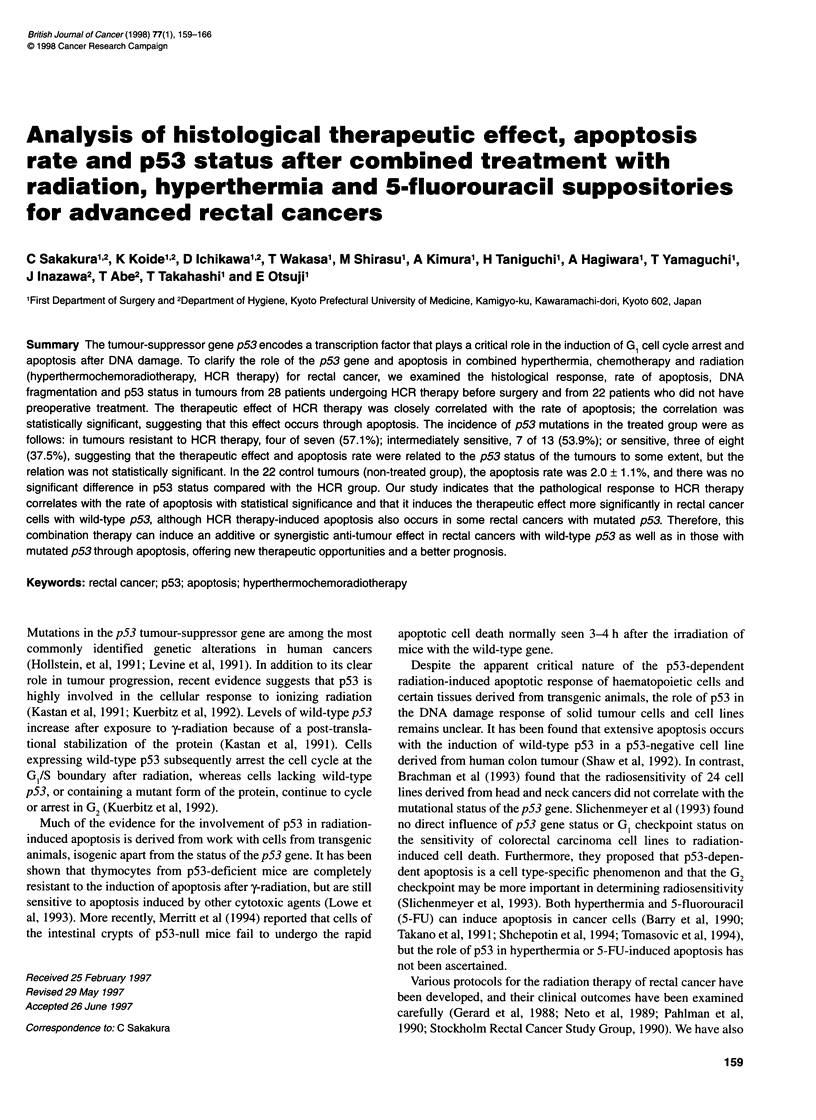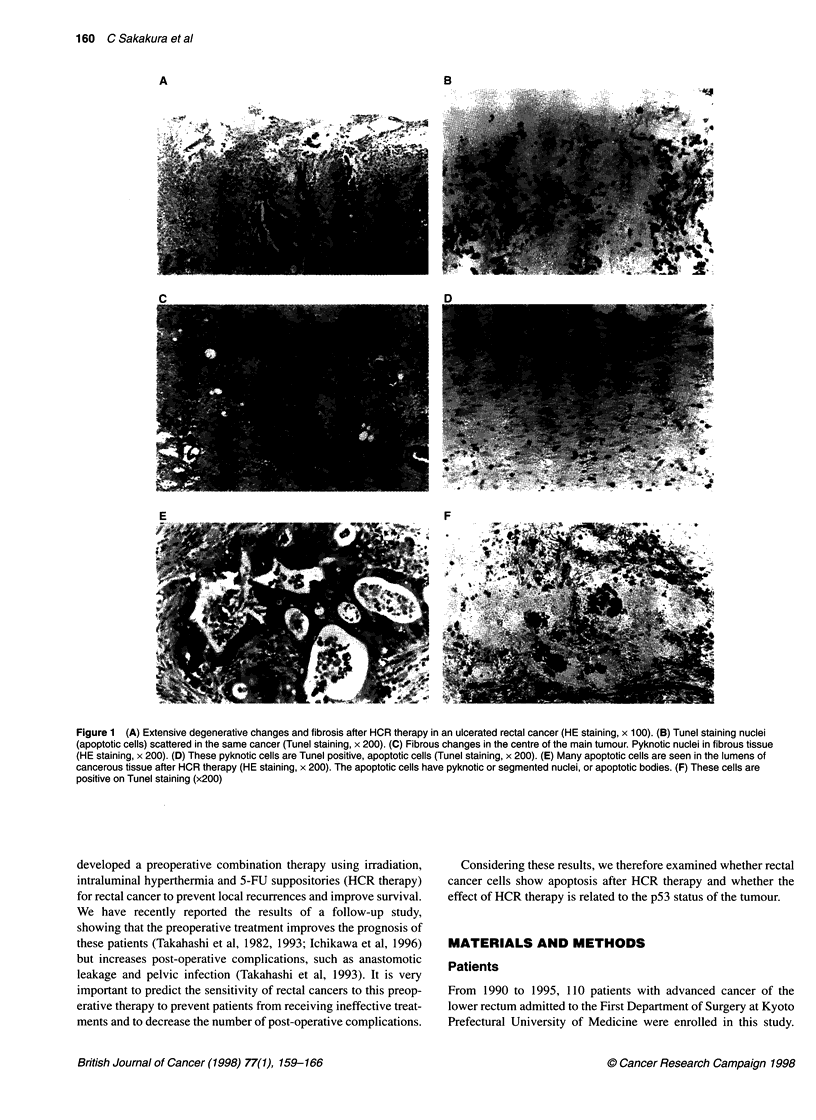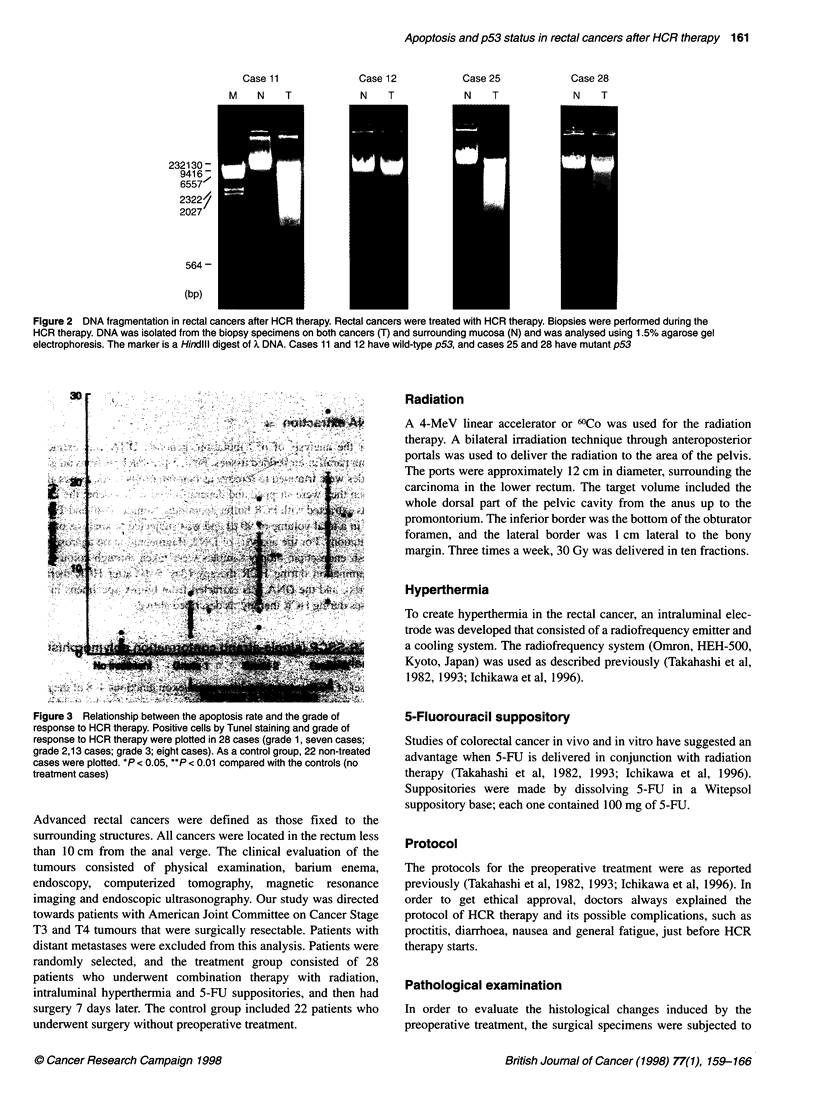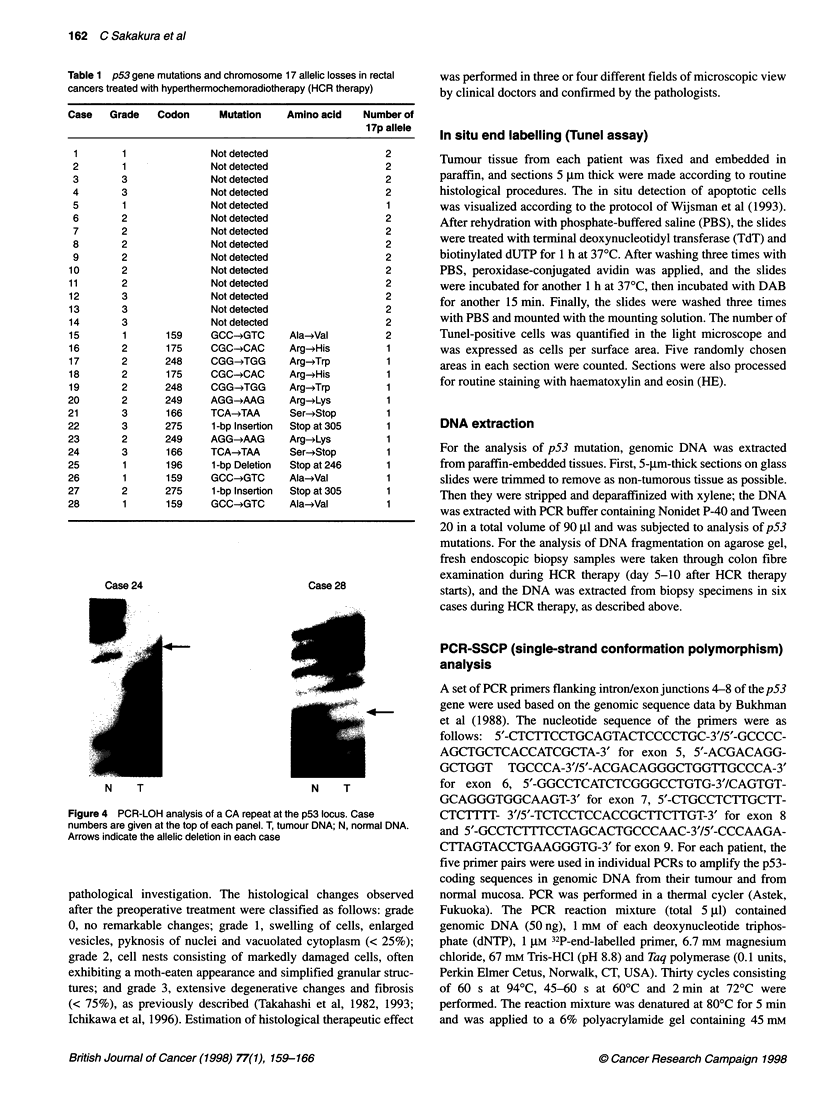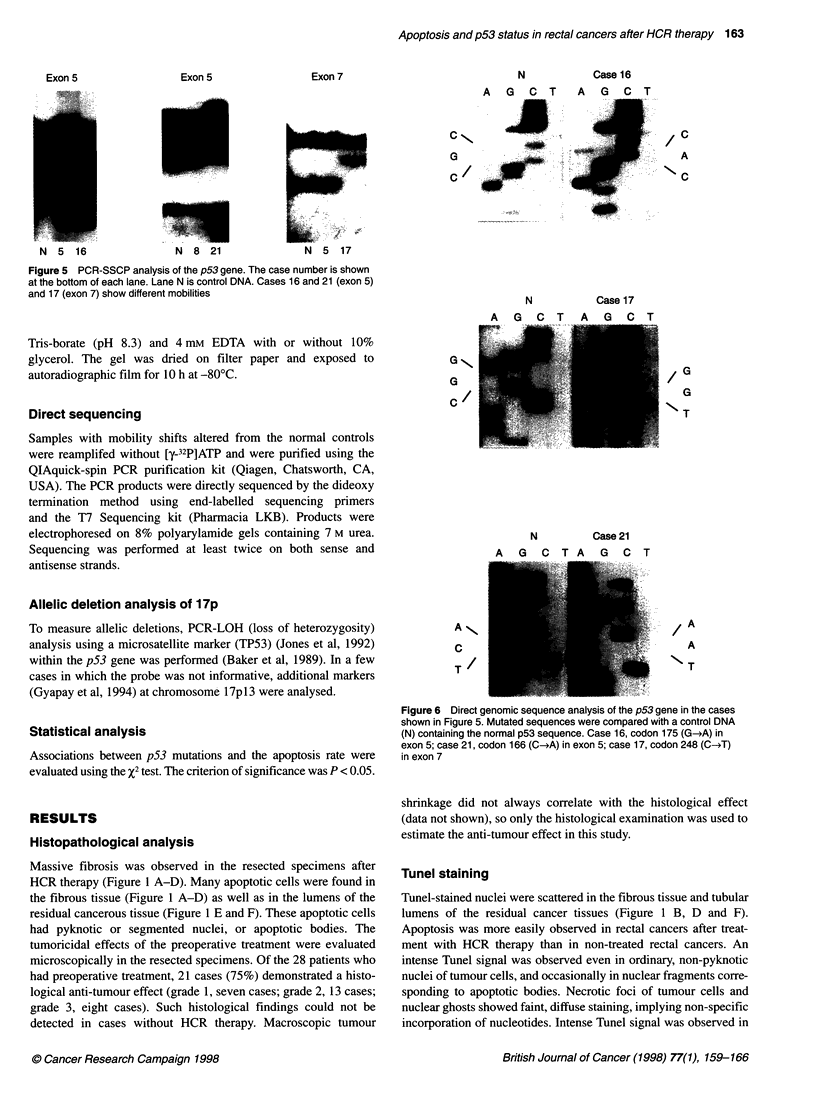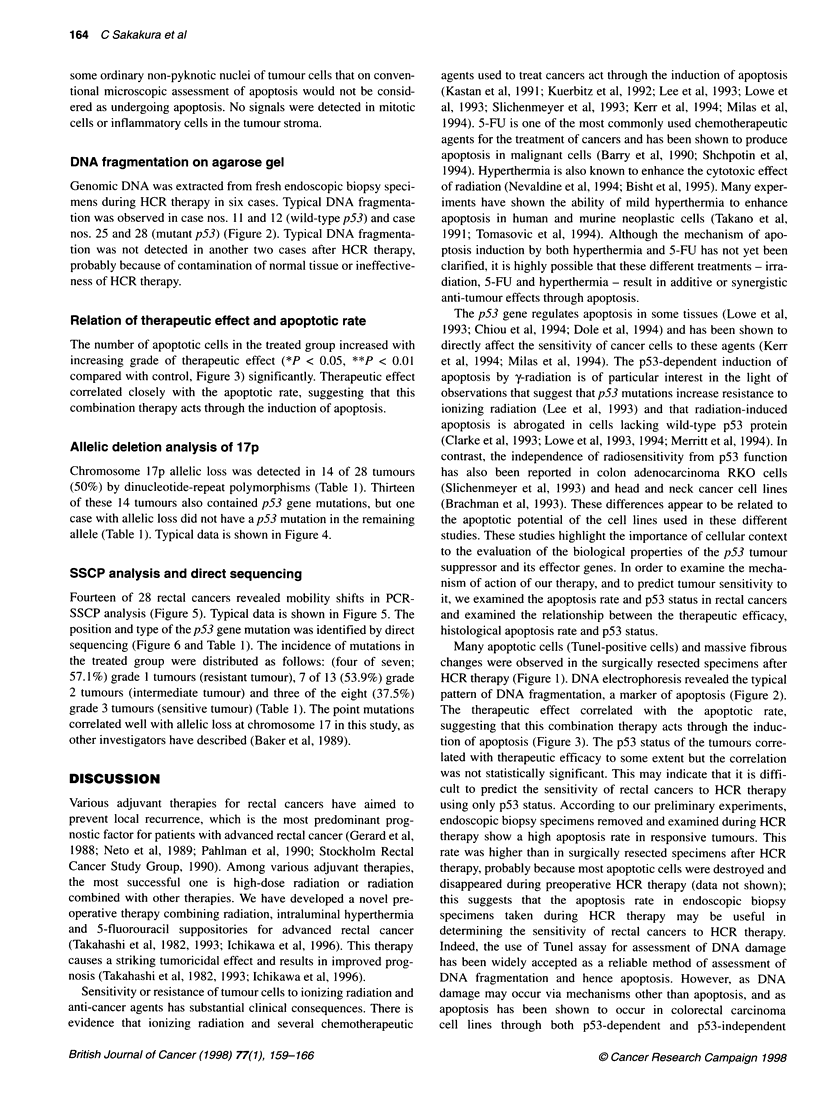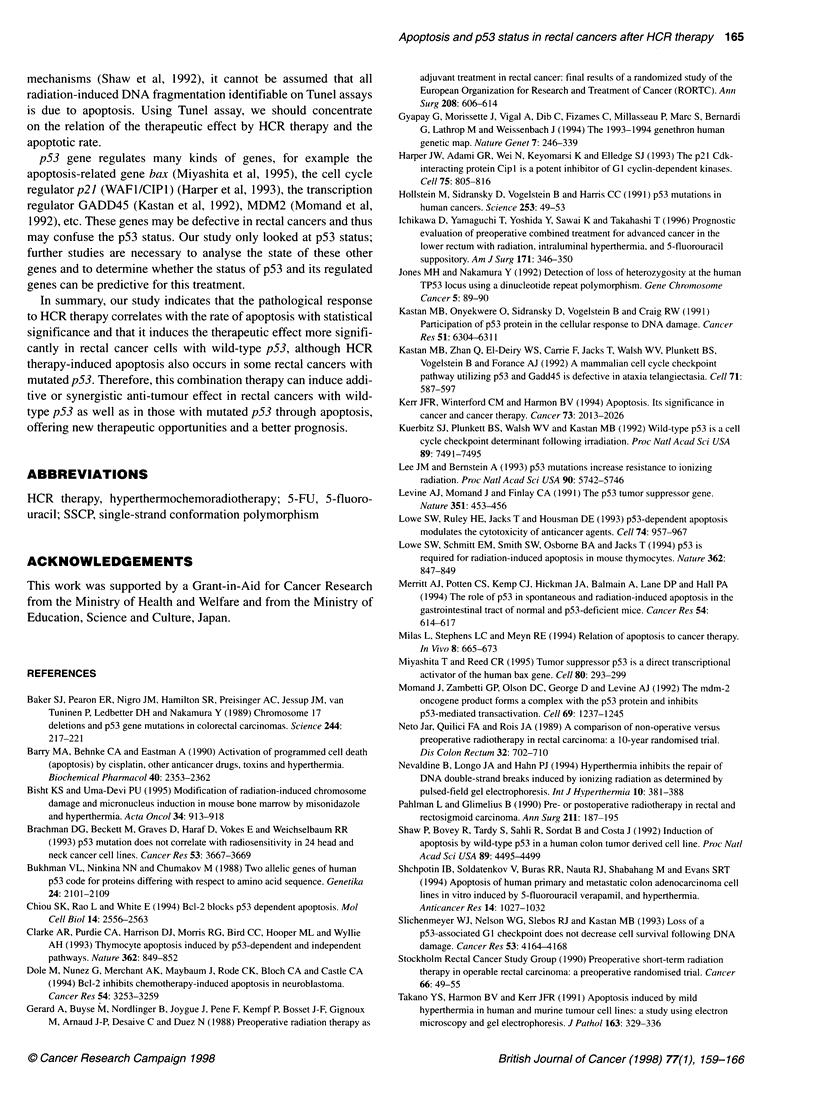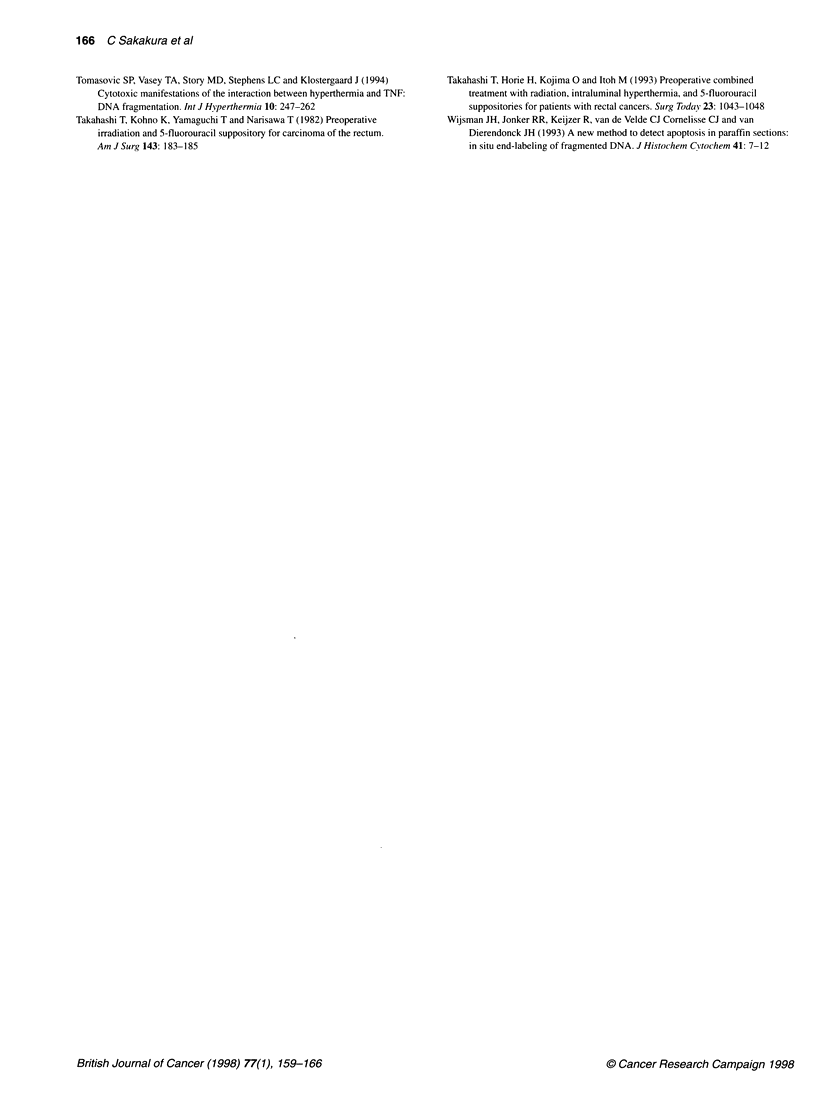# Analysis of histological therapeutic effect, apoptosis rate and p53 status after combined treatment with radiation, hyperthermia and 5-fluorouracil suppositories for advanced rectal cancers.

**DOI:** 10.1038/bjc.1998.25

**Published:** 1998

**Authors:** C. Sakakura, K. Koide, D. Ichikawa, T. Wakasa, M. Shirasu, A. Kimura, H. Taniguchi, A. Hagiwara, T. Yamaguchi, J. Inazawa, T. Abe, T. Takahashi, E. Otsuji

**Affiliations:** First Department of Surgery, Kyoto Prefectural University of Medicine, Japan.

## Abstract

**Images:**


British Journal of Cancer (1998) 77(1), 159–166
© 1998 Cancer Research Campaign

### Analysis of histological therapeutic effect, apoptosis rate and p53 status after combined treatment with radiation, hyperthermia and 5-fluorouracil suppositories for advanced rectal cancers

C Sakakura[1,2], K Koide[1,2], D Ichikawa[1,2], T Wakasa[1], M Shirasu[1], A Kimura[1], H Taniguchi[1], A Hagiwara[1], T Yamaguchi[1], J Inazawa[2], T Abe[2], T Takahashi[1] and E Otsuji[1]

[1]First Department of Surgery and [2]Department of Hygiene, Kyoto Prefectural University of Medicine, Kamigyo-ku, Kawaramachi-dori, Kyoto 602, Japan

**Summary** The tumour-suppressor gene *p53* encodes a transcription factor that plays a critical role in the induction of $G_1$ cell cycle arrest and apoptosis after DNA damage. To clarify the role of the *p53* gene and apoptosis in combined hyperthermia, chemotherapy and radiation (hyperthermochemoradiotherapy, HCR therapy) for rectal cancer, we examined the histological response, rate of apoptosis, DNA fragmentation and p53 status in tumours from 28 patients undergoing HCR therapy before surgery and from 22 patients who did not have preoperative treatment. The therapeutic effect of HCR therapy was closely correlated with the rate of apoptosis; the correlation was statistically significant, suggesting that this effect occurs through apoptosis. The incidence of *p53* mutations in the treated group were as follows: in tumours resistant to HCR therapy, four of seven (57.1%); intermediately sensitive, 7 of 13 (53.9%); or sensitive, three of eight (37.5%), suggesting that the therapeutic effect and apoptosis rate were related to the *p53* status of the tumours to some extent, but the relation was not statistically significant. In the 22 control tumours (non-treated group), the apoptosis rate was 2.0 ± 1.1%, and there was no significant difference in p53 status compared with the HCR group. Our study indicates that the pathological response to HCR therapy correlates with the rate of apoptosis with statistical significance and that it induces the therapeutic effect more significantly in rectal cancer cells with wild-type *p53*, although HCR therapy-induced apoptosis also occurs in some rectal cancers with mutated *p53*. Therefore, this combination therapy can induce an additive or synergistic anti-tumour effect in rectal cancers with wild-type *p53* as well as in those with mutated *p53* through apoptosis, offering new therapeutic opportunities and a better prognosis.

**Keywords:** rectal cancer; p53; apoptosis; hyperthermochemoradiotherapy

Mutations in the *p53* tumour-suppressor gene are among the most commonly identified genetic alterations in human cancers (Hollstein, et al, 1991; Levine et al, 1991). In addition to its clear role in tumour progression, recent evidence suggests that p53 is highly involved in the cellular response to ionizing radiation (Kastan et al, 1991; Kuerbitz et al, 1992). Levels of wild-type *p53* increase after exposure to γ-radiation because of a post-translational stabilization of the protein (Kastan et al, 1991). Cells expressing wild-type p53 subsequently arrest the cell cycle at the $G_1$/S boundary after radiation, whereas cells lacking wild-type *p53*, or containing a mutant form of the protein, continue to cycle or arrest in $G_2$ (Kuerbitz et al, 1992).

Much of the evidence for the involvement of p53 in radiation-induced apoptosis is derived from work with cells from transgenic animals, isogenic apart from the status of the *p53* gene. It has been shown that thymocytes from p53-deficient mice are completely resistant to the induction of apoptosis after γ-radiation, but are still sensitive to apoptosis induced by other cytotoxic agents (Lowe et al, 1993). More recently, Merritt et al (1994) reported that cells of the intestinal crypts of p53-null mice fail to undergo the rapid apoptotic cell death normally seen 3–4 h after the irradiation of mice with the wild-type gene.

Despite the apparent critical nature of the p53-dependent radiation-induced apoptotic response of haematopoietic cells and certain tissues derived from transgenic animals, the role of p53 in the DNA damage response of solid tumour cells and cell lines remains unclear. It has been found that extensive apoptosis occurs with the induction of wild-type p53 in a p53-negative cell line derived from human colon tumour (Shaw et al, 1992). In contrast, Brachman et al (1993) found that the radiosensitivity of 24 cell lines derived from head and neck cancers did not correlate with the mutational status of the *p53* gene. Slichenmeyer et al (1993) found no direct influence of *p53* gene status or $G_1$ checkpoint status on the sensitivity of colorectal carcinoma cell lines to radiation-induced cell death. Furthermore, they proposed that p53-dependent apoptosis is a cell type-specific phenomenon and that the $G_2$ checkpoint may be more important in determining radiosensitivity (Slichenmeyer et al, 1993). Both hyperthermia and 5-fluorouracil (5-FU) can induce apoptosis in cancer cells (Barry et al, 1990; Takano et al, 1991; Shchepotin et al, 1994; Tomasovic et al, 1994), but the role of p53 in hyperthermia or 5-FU-induced apoptosis has not been ascertained.

Various protocols for the radiation therapy of rectal cancer have been developed, and their clinical outcomes have been examined carefully (Gerard et al, 1988; Neto et al, 1989; Pahlman et al, 1990; Stockholm Rectal Cancer Study Group, 1990). We have also

*Received 25 February 1997*
*Revised 29 May 1997*
*Accepted 26 June 1997*

*Correspondence to:* C Sakakura

**Figure 1** (**A**) Extensive degenerative changes and fibrosis after HCR therapy in an ulcerated rectal cancer (HE staining, × 100). (**B**) Tunel staining nuclei (apoptotic cells) scattered in the same cancer (Tunel staining, × 200). (**C**) Fibrous changes in the centre of the main tumour. Pyknotic nuclei in fibrous tissue (HE staining, × 200). (**D**) These pyknotic cells are Tunel positive, apoptotic cells (Tunel staining, × 200). (**E**) Many apoptotic cells are seen in the lumens of cancerous tissue after HCR therapy (HE staining, × 200). The apoptotic cells have pyknotic or segmented nuclei, or apoptotic bodies. (**F**) These cells are positive on Tunel staining (×200)

developed a preoperative combination therapy using irradiation, intraluminal hyperthermia and 5-FU suppositories (HCR therapy) for rectal cancer to prevent local recurrences and improve survival. We have recently reported the results of a follow-up study, showing that the preoperative treatment improves the prognosis of these patients (Takahashi et al, 1982, 1993; Ichikawa et al, 1996) but increases post-operative complications, such as anastomotic leakage and pelvic infection (Takahashi et al, 1993). It is very important to predict the sensitivity of rectal cancers to this preoperative therapy to prevent patients from receiving ineffective treatments and to decrease the number of post-operative complications.

Considering these results, we therefore examined whether rectal cancer cells show apoptosis after HCR therapy and whether the effect of HCR therapy is related to the p53 status of the tumour.

### MATERIALS AND METHODS

### Patients

From 1990 to 1995, 110 patients with advanced cancer of the lower rectum admitted to the First Department of Surgery at Kyoto Prefectural University of Medicine were enrolled in this study.

© Cancer Research Campaign 1998

**Figure 2** DNA fragmentation in rectal cancers after HCR therapy. Rectal cancers were treated with HCR therapy. Biopsies were performed during the HCR therapy. DNA was isolated from the biopsy specimens on both cancers (T) and surrounding mucosa (N) and was analysed using 1.5% agarose gel electrophoresis. The marker is a *Hin*dIII digest of λ DNA. Cases 11 and 12 have wild-type *p53*, and cases 25 and 28 have mutant *p53*

**Figure 3** Relationship between the apoptosis rate and the grade of response to HCR therapy. Positive cells by Tunel staining and grade of response to HCR therapy were plotted in 28 cases (grade 1, seven cases; grade 2,13 cases; grade 3; eight cases). As a control group, 22 non-treated cases were plotted. $^*P < 0.05$, $^{**}P < 0.01$ compared with the controls (no treatment cases)

Advanced rectal cancers were defined as those fixed to the surrounding structures. All cancers were located in the rectum less than 10 cm from the anal verge. The clinical evaluation of the tumours consisted of physical examination, barium enema, endoscopy, computerized tomography, magnetic resonance imaging and endoscopic ultrasonography. Our study was directed towards patients with American Joint Committee on Cancer Stage T3 and T4 tumours that were surgically resectable. Patients with distant metastases were excluded from this analysis. Patients were randomly selected, and the treatment group consisted of 28 patients who underwent combination therapy with radiation, intraluminal hyperthermia and 5-FU suppositories, and then had surgery 7 days later. The control group included 22 patients who underwent surgery without preoperative treatment.

### Radiation

A 4-MeV linear accelerator or $^{60}$Co was used for the radiation therapy. A bilateral irradiation technique through anteroposterior portals was used to deliver the radiation to the area of the pelvis. The ports were approximately 12 cm in diameter, surrounding the carcinoma in the lower rectum. The target volume included the whole dorsal part of the pelvic cavity from the anus up to the promontorium. The inferior border was the bottom of the obturator foramen, and the lateral border was 1 cm lateral to the bony margin. Three times a week, 30 Gy was delivered in ten fractions.

### Hyperthermia

To create hyperthermia in the rectal cancer, an intraluminal electrode was developed that consisted of a radiofrequency emitter and a cooling system. The radiofrequency system (Omron, HEH-500, Kyoto, Japan) was used as described previously (Takahashi et al, 1982, 1993; Ichikawa et al, 1996).

### 5-Fluorouracil suppository

Studies of colorectal cancer in vivo and in vitro have suggested an advantage when 5-FU is delivered in conjunction with radiation therapy (Takahashi et al, 1982, 1993; Ichikawa et al, 1996). Suppositories were made by dissolving 5-FU in a Witepsol suppository base; each one contained 100 mg of 5-FU.

### Protocol

The protocols for the preoperative treatment were as reported previously (Takahashi et al, 1982, 1993; Ichikawa et al, 1996). In order to get ethical approval, doctors always explained the protocol of HCR therapy and its possible complications, such as proctitis, diarrhoea, nausea and general fatigue, just before HCR therapy starts.

### Pathological examination

In order to evaluate the histological changes induced by the preoperative treatment, the surgical specimens were subjected to

© Cancer Research Campaign 1998

**Table 1** *p53* gene mutations and chromosome 17 allelic losses in rectal cancers treated with hyperthermochemoradiotherapy (HCR therapy)

| Case | Grade | Codon | Mutation | Amino acid | Number of 17p allele |
|---|---|---|---|---|---|
| 1 | 1 | | Not detected | | 2 |
| 2 | 1 | | Not detected | | 2 |
| 3 | 3 | | Not detected | | 2 |
| 4 | 3 | | Not detected | | 2 |
| 5 | 1 | | Not detected | | 1 |
| 6 | 2 | | Not detected | | 2 |
| 7 | 2 | | Not detected | | 2 |
| 8 | 2 | | Not detected | | 2 |
| 9 | 2 | | Not detected | | 2 |
| 10 | 2 | | Not detected | | 2 |
| 11 | 2 | | Not detected | | 2 |
| 12 | 3 | | Not detected | | 2 |
| 13 | 3 | | Not detected | | 2 |
| 14 | 3 | | Not detected | | 2 |
| 15 | 1 | 159 | GCC→GTC | Ala→Val | 2 |
| 16 | 2 | 175 | CGC→CAC | Arg→His | 1 |
| 17 | 2 | 248 | CGG→TGG | Arg→Trp | 1 |
| 18 | 2 | 175 | CGC→CAC | Arg→His | 1 |
| 19 | 2 | 248 | CGG→TGG | Arg→Trp | 1 |
| 20 | 2 | 249 | AGG→AAG | Arg→Lys | 1 |
| 21 | 3 | 166 | TCA→TAA | Ser→Stop | 1 |
| 22 | 3 | 275 | 1-bp Insertion | Stop at 305 | 1 |
| 23 | 2 | 249 | AGG→AAG | Arg→Lys | 1 |
| 24 | 3 | 166 | TCA→TAA | Ser→Stop | 1 |
| 25 | 1 | 196 | 1-bp Deletion | Stop at 246 | 1 |
| 26 | 1 | 159 | GCC→GTC | Ala→Val | 1 |
| 27 | 2 | 275 | 1-bp Insertion | Stop at 305 | 1 |
| 28 | 1 | 159 | GCC→GTC | Ala→Val | 1 |

**Figure 4** PCR-LOH analysis of a CA repeat at the p53 locus. Case numbers are given at the top of each panel. T, tumour DNA; N, normal DNA. Arrows indicate the allelic deletion in each case pathological investigation. The histological changes observed after the preoperative treatment were classified as follows: grade 0, no remarkable changes; grade 1, swelling of cells, enlarged vesicles, pyknosis of nuclei and vacuolated cytoplasm (< 25%); grade 2, cell nests consisting of markedly damaged cells, often exhibiting a moth-eaten appearance and simplified granular structures; and grade 3, extensive degenerative changes and fibrosis (< 75%), as previously described (Takahashi et al, 1982, 1993; Ichikawa et al, 1996). Estimation of histological therapeutic effect was performed in three or four different fields of microscopic view by clinical doctors and confirmed by the pathologists.

### In situ end labelling (Tunel assay)

Tumour tissue from each patient was fixed and embedded in paraffin, and sections 5 μm thick were made according to routine histological procedures. The in situ detection of apoptotic cells was visualized according to the protocol of Wijsman et al (1993). After rehydration with phosphate-buffered saline (PBS), the slides were treated with terminal deoxynucleotidyl transferase (TdT) and biotinylated dUTP for 1 h at 37°C. After washing three times with PBS, peroxidase-conjugated avidin was applied, and the slides were incubated for another 1 h at 37°C, then incubated with DAB for another 15 min. Finally, the slides were washed three times with PBS and mounted with the mounting solution. The number of Tunel-positive cells was quantified in the light microscope and was expressed as cells per surface area. Five randomly chosen areas in each section were counted. Sections were also processed for routine staining with haematoxylin and eosin (HE).

### DNA extraction

For the analysis of *p53* mutation, genomic DNA was extracted from paraffin-embedded tissues. First, 5-μm-thick sections on glass slides were trimmed to remove as non-tumorous tissue as possible. Then they were stripped and deparaffinized with xylene; the DNA was extracted with PCR buffer containing Nonidet P-40 and Tween 20 in a total volume of 90 μl and was subjected to analysis of *p53* mutations. For the analysis of DNA fragmentation on agarose gel, fresh endoscopic biopsy samples were taken through colon fibre examination during HCR therapy (day 5–10 after HCR therapy starts), and the DNA was extracted from biopsy specimens in six cases during HCR therapy, as described above.

### PCR-SSCP (single-strand conformation polymorphism) analysis

A set of PCR primers flanking intron/exon junctions 4–8 of the *p53* gene were used based on the genomic sequence data by Bukhman et al (1988). The nucleotide sequence of the primers were as follows: 5′-CTCTTCCTGCAGTACTCCCCTGC-3′/5′-GCCCC-AGCTGCTCACCATCGCTA-3′ for exon 5, 5′-ACGACAGG-GCTGGT TGCCCA-3′/5′-ACGACAGGGCTGGTTGCCCA-3′ for exon 6, 5′-GGCCTCATCTCGGGCCTGTG-3′/CAGTGT-GCAGGGTGGCAAGT-3′ for exon 7, 5′-CTGCCTCTTGCTT-CTCTTTT- 3′/5′-TCTCCTCCACCGCTTCTTGT-3′ for exon 8 and 5′-GCCTCTTTCCTAGCACTGCCCAAC-3′/5′-CCCAAGA-CTTAGTACCTGAAGGGTG-3′ for exon 9. For each patient, the five primer pairs were used in individual PCRs to amplify the p53-coding sequences in genomic DNA from their tumour and from normal mucosa. PCR was performed in a thermal cycler (Astek, Fukuoka). The PCR reaction mixture (total 5 μl) contained genomic DNA (50 ng), 1 mM of each deoxynucleotide triphosphate (dNTP), 1 μM $^{32}$P-end-labelled primer, 6.7 mM magnesium chloride, 67 mM Tris-HCl (pH 8.8) and *Taq* polymerase (0.1 units, Perkin Elmer Cetus, Norwalk, CT, USA). Thirty cycles consisting of 60 s at 94°C, 45–60 s at 60°C and 2 min at 72°C were performed. The reaction mixture was denatured at 80°C for 5 min and was applied to a 6% polyacrylamide gel containing 45 mM

© Cancer Research Campaign 1998

**Figure 5** PCR-SSCP analysis of the *p53* gene. The case number is shown at the bottom of each lane. Lane N is control DNA. Cases 16 and 21 (exon 5) and 17 (exon 7) show different mobilities

**Figure 6** Direct genomic sequence analysis of the *p53* gene in the cases shown in Figure 5. Mutated sequences were compared with a control DNA (N) containing the normal p53 sequence. Case 16, codon 175 (G→A) in exon 5; case 21, codon 166 (C→A) in exon 5; case 17, codon 248 (C→T) in exon 7

Tris-borate (pH 8.3) and 4 mM EDTA with or without 10% glycerol. The gel was dried on filter paper and exposed to autoradiographic film for 10 h at –80°C.

### Direct sequencing

Samples with mobility shifts altered from the normal controls were reamplifed without [γ-$^{32}$P]ATP and were purified using the QIAquick-spin PCR purification kit (Qiagen, Chatsworth, CA, USA). The PCR products were directly sequenced by the dideoxy termination method using end-labelled sequencing primers and the T7 Sequencing kit (Pharmacia LKB). Products were electrophoresed on 8% polyarylamide gels containing 7 M urea. Sequencing was performed at least twice on both sense and antisense strands.

### Allelic deletion analysis of 17p

To measure allelic deletions, PCR-LOH (loss of heterozygosity) analysis using a microsatellite marker (TP53) (Jones et al, 1992) within the *p53* gene was performed (Baker et al, 1989). In a few cases in which the probe was not informative, additional markers (Gyapay et al, 1994) at chromosome 17p13 were analysed.

### Statistical analysis

Associations between *p53* mutations and the apoptosis rate were evaluated using the $\chi^2$ test. The criterion of significance was $P < 0.05$.

### RESULTS

### Histopathological analysis

Massive fibrosis was observed in the resected specimens after HCR therapy (Figure 1 A–D). Many apoptotic cells were found in the fibrous tissue (Figure 1 A–D) as well as in the lumens of the residual cancerous tissue (Figure 1 E and F). These apoptotic cells had pyknotic or segmented nuclei, or apoptotic bodies. The tumoricidal effects of the preoperative treatment were evaluated microscopically in the resected specimens. Of the 28 patients who had preoperative treatment, 21 cases (75%) demonstrated a histological anti-tumour effect (grade 1, seven cases; grade 2, 13 cases; grade 3, eight cases). Such histological findings could not be detected in cases without HCR therapy. Macroscopic tumour shrinkage did not always correlate with the histological effect (data not shown), so only the histological examination was used to estimate the anti-tumour effect in this study.

### Tunel staining

Tunel-stained nuclei were scattered in the fibrous tissue and tubular lumens of the residual cancer tissues (Figure 1 B, D and F). Apoptosis was more easily observed in rectal cancers after treatment with HCR therapy than in non-treated rectal cancers. An intense Tunel signal was observed even in ordinary, non-pyknotic nuclei of tumour cells, and occasionally in nuclear fragments corresponding to apoptotic bodies. Necrotic foci of tumour cells and nuclear ghosts showed faint, diffuse staining, implying non-specific incorporation of nucleotides. Intense Tunel signal was observed in

© Cancer Research Campaign 1998

some ordinary non-pyknotic nuclei of tumour cells that on conventional microscopic assessment of apoptosis would not be considered as undergoing apoptosis. No signals were detected in mitotic cells or inflammatory cells in the tumour stroma.

### DNA fragmentation on agarose gel

Genomic DNA was extracted from fresh endoscopic biopsy specimens during HCR therapy in six cases. Typical DNA fragmentation was observed in case nos. 11 and 12 (wild-type *p53*) and case nos. 25 and 28 (mutant *p53*) (Figure 2). Typical DNA fragmentation was not detected in another two cases after HCR therapy, probably because of contamination of normal tissue or ineffectiveness of HCR therapy.

### Relation of therapeutic effect and apoptotic rate

The number of apoptotic cells in the treated group increased with increasing grade of therapeutic effect ($^{*}P < 0.05$, $^{**}P < 0.01$ compared with control, Figure 3) significantly. Therapeutic effect correlated closely with the apoptotic rate, suggesting that this combination therapy acts through the induction of apoptosis.

### Allelic deletion analysis of 17p

Chromosome 17p allelic loss was detected in 14 of 28 tumours (50%) by dinucleotide-repeat polymorphisms (Table 1). Thirteen of these 14 tumours also contained *p53* gene mutations, but one case with allelic loss did not have a *p53* mutation in the remaining allele (Table 1). Typical data is shown in Figure 4.

### SSCP analysis and direct sequencing

Fourteen of 28 rectal cancers revealed mobility shifts in PCR-SSCP analysis (Figure 5). Typical data is shown in Figure 5. The position and type of the *p53* gene mutation was identified by direct sequencing (Figure 6 and Table 1). The incidence of mutations in the treated group were distributed as follows: (four of seven; 57.1%) grade 1 tumours (resistant tumour), 7 of 13 (53.9%) grade 2 tumours (intermediate tumour) and three of the eight (37.5%) grade 3 tumours (sensitive tumour) (Table 1). The point mutations correlated well with allelic loss at chromosome 17 in this study, as other investigators have described (Baker et al, 1989).

### DISCUSSION

Various adjuvant therapies for rectal cancers have aimed to prevent local recurrence, which is the most predominant prognostic factor for patients with advanced rectal cancer (Gerard et al, 1988; Neto et al, 1989; Pahlman et al, 1990; Stockholm Rectal Cancer Study Group, 1990). Among various adjuvant therapies, the most successful one is high-dose radiation or radiation combined with other therapies. We have developed a novel preoperative therapy combining radiation, intraluminal hyperthermia and 5-fluorouracil suppositories for advanced rectal cancer (Takahashi et al, 1982, 1993; Ichikawa et al, 1996). This therapy causes a striking tumoricidal effect and results in improved prognosis (Takahashi et al, 1982, 1993; Ichikawa et al, 1996).

Sensitivity or resistance of tumour cells to ionizing radiation and anti-cancer agents has substantial clinical consequences. There is evidence that ionizing radiation and several chemotherapeutic agents used to treat cancers act through the induction of apoptosis (Kastan et al, 1991; Kuerbitz et al, 1992; Lee et al, 1993; Lowe et al, 1993; Slichenmeyer et al, 1993; Kerr et al, 1994; Milas et al, 1994). 5-FU is one of the most commonly used chemotherapeutic agents for the treatment of cancers and has been shown to produce apoptosis in malignant cells (Barry et al, 1990; Shchpotin et al, 1994). Hyperthermia is also known to enhance the cytotoxic effect of radiation (Nevaldine et al, 1994; Bisht et al, 1995). Many experiments have shown the ability of mild hyperthermia to enhance apoptosis in human and murine neoplastic cells (Takano et al, 1991; Tomasovic et al, 1994). Although the mechanism of apoptosis induction by both hyperthermia and 5-FU has not yet been clarified, it is highly possible that these different treatments – irradiation, 5-FU and hyperthermia – result in additive or synergistic anti-tumour effects through apoptosis.

The *p53* gene regulates apoptosis in some tissues (Lowe et al, 1993; Chiou et al, 1994; Dole et al, 1994) and has been shown to directly affect the sensitivity of cancer cells to these agents (Kerr et al, 1994; Milas et al, 1994). The p53-dependent induction of apoptosis by $\gamma$-radiation is of particular interest in the light of observations that suggest that *p53* mutations increase resistance to ionizing radiation (Lee et al, 1993) and that radiation-induced apoptosis is abrogated in cells lacking wild-type p53 protein (Clarke et al, 1993; Lowe et al, 1993, 1994; Merritt et al, 1994). In contrast, the independence of radiosensitivity from p53 function has also been reported in colon adenocarcinoma RKO cells (Slichenmeyer et al, 1993) and head and neck cancer cell lines (Brachman et al, 1993). These differences appear to be related to the apoptotic potential of the cell lines used in these different studies. These studies highlight the importance of cellular context to the evaluation of the biological properties of the *p53* tumour suppressor and its effector genes. In order to examine the mechanism of action of our therapy, and to predict tumour sensitivity to it, we examined the apoptosis rate and p53 status in rectal cancers and examined the relationship between the therapeutic efficacy, histological apoptosis rate and p53 status.

Many apoptotic cells (Tunel-positive cells) and massive fibrous changes were observed in the surgically resected specimens after HCR therapy (Figure 1). DNA electrophoresis revealed the typical pattern of DNA fragmentation, a marker of apoptosis (Figure 2). The therapeutic effect correlated with the apoptotic rate, suggesting that this combination therapy acts through the induction of apoptosis (Figure 3). The p53 status of the tumours correlated with therapeutic efficacy to some extent but the correlation was not statistically significant. This may indicate that it is difficult to predict the sensitivity of rectal cancers to HCR therapy using only p53 status. According to our preliminary experiments, endoscopic biopsy specimens removed and examined during HCR therapy show a high apoptosis rate in responsive tumours. This rate was higher than in surgically resected specimens after HCR therapy, probably because most apoptotic cells were destroyed and disappeared during preoperative HCR therapy (data not shown); this suggests that the apoptosis rate in endoscopic biopsy specimens taken during HCR therapy may be useful in determining the sensitivity of rectal cancers to HCR therapy. Indeed, the use of Tunel assay for assessment of DNA damage has been widely accepted as a reliable method of assessment of DNA fragmentation and hence apoptosis. However, as DNA damage may occur via mechanisms other than apoptosis, and as apoptosis has been shown to occur in colorectal carcinoma cell lines through both p53-dependent and p53-independent

© Cancer Research Campaign 1998

mechanisms (Shaw et al, 1992), it cannot be assumed that all radiation-induced DNA fragmentation identifiable on Tunel assays is due to apoptosis. Using Tunel assay, we should concentrate on the relation of the therapeutic effect by HCR therapy and the apoptotic rate.

*p53* gene regulates many kinds of genes, for example the apoptosis-related gene *bax* (Miyashita et al, 1995), the cell cycle regulator *p21* (WAF1/CIP1) (Harper et al, 1993), the transcription regulator GADD45 (Kastan et al, 1992), MDM2 (Momand et al, 1992), etc. These genes may be defective in rectal cancers and thus may confuse the p53 status. Our study only looked at p53 status; further studies are necessary to analyse the state of these other genes and to determine whether the status of p53 and its regulated genes can be predictive for this treatment.

In summary, our study indicates that the pathological response to HCR therapy correlates with the rate of apoptosis with statistical significance and that it induces the therapeutic effect more significantly in rectal cancer cells with wild-type *p53*, although HCR therapy-induced apoptosis also occurs in some rectal cancers with mutated *p53*. Therefore, this combination therapy can induce additive or synergistic anti-tumour effect in rectal cancers with wild-type *p53* as well as in those with mutated *p53* through apoptosis, offering new therapeutic opportunities and a better prognosis.

### ABBREVIATIONS

HCR therapy, hyperthermochemoradiotherapy; 5-FU, 5-fluorouracil; SSCP, single-strand conformation polymorphism

### ACKNOWLEDGEMENTS

This work was supported by a Grant-in-Aid for Cancer Research from the Ministry of Health and Welfare and from the Ministry of Education, Science and Culture, Japan.

### REFERENCES

Baker SJ, Pearon ER, Nigro JM, Hamilton SR, Preisinger AC, Jessup JM, van Tuninen P, Ledbetter DH and Nakamura Y (1989) Chromosome 17 deletions and p53 gene mutations in colorectal carcinomas. *Science* **244**: 217–221

Barry MA, Behnke CA and Eastman A (1990) Activation of programmed cell death (apoptosis) by cisplatin, other anticancer drugs, toxins and hyperthermia. *Biochemical Pharmacol* **40**: 2353–2362

Bisht KS and Uma-Devi PU (1995) Modification of radiation-induced chromosome damage and micronucleus induction in mouse bone marrow by misonidazole and hyperthermia. *Acta Oncol* **34**: 913–918

Brachman DG, Beckett M, Graves D, Haraf D, Vokes E and Weichselbaum RR (1993) p53 mutation does not correlate with radiosensitivity in 24 head and neck cancer cell lines. *Cancer Res* **53**: 3667–3669

Bukhman VL, Ninkina NN and Chumakov M (1988) Two allelic genes of human p53 code for proteins differing with respect to amino acid sequence. *Genetika* **24**: 2101–2109

Chiou SK, Rao L and White E (1994) Bcl-2 blocks p53 dependent apoptosis. *Mol Cell Biol* **14**: 2556–2563

Clarke AR, Purdie CA, Harrison DJ, Morris RG, Bird CC, Hooper ML and Wyllie AH (1993) Thymocyte apoptosis induced by p53-dependent and independent pathways. *Nature* **362**: 849–852

Dole M, Nunez G, Merchant AK, Maybaum J, Rode CK, Bloch CA and Castle CA (1994) Bcl-2 inhibits chemotherapy-induced apoptosis in neuroblastoma. *Cancer Res* **54**: 3253–3259

Gerard A, Buyse M, Nordlinger B, Joygue J, Pene F, Kempf P, Bosset J-F, Gignoux M, Arnaud J-P, Desaive C and Duez N (1988) Preoperative radiation therapy as adjuvant treatment in rectal cancer: final results of a randomized study of the European Organization for Research and Treatment of Cancer (RORTC). *Ann Surg* **208**: 606–614

Gyapay G, Morissette J, Vigal A, Dib C, Fizames C, Millasseau P, Marc S, Bernardi G, Lathrop M and Weissenbach J (1994) The 1993–1994 genethron human genetic map. *Nature Genet* **7**: 246–339

Harper JW, Adami GR, Wei N, Keyomarsi K and Elledge SJ (1993) The p21 Cdk-interacting protein Cip1 is a potent inhibitor of G1 cyclin-dependent kinases. *Cell* **75**: 805–816

Hollstein M, Sidransky D, Vogelstein B and Harris CC (1991) p53 mutations in human cancers. *Science* **253**: 49–53

Ichikawa D, Yamaguchi T, Yoshida Y, Sawai K and Takahashi T (1996) Prognostic evaluation of preoperative combined treatment for advanced cancer in the lower rectum with radiation, intraluminal hyperthermia, and 5-fluorouracil suppository. *Am J Surg* **171**: 346–350

Jones MH and Nakamura Y (1992) Detection of loss of heterozygosity at the human TP53 locus using a dinucleotide repeat polymorphism. *Gene Chromosome Cancer* **5**: 89–90

Kastan MB, Onyekwere O, Sidransky D, Vogelstein B and Craig RW (1991) Participation of p53 protein in the cellular response to DNA damage. *Cancer Res* **51**: 6304–6311

Kastan MB, Zhan Q, El-Deiry WS, Carrie F, Jacks T, Walsh WV, Plunkett BS, Vogelstein B and Forance AJ (1992) A mammalian cell cycle checkpoint pathway utilizing p53 and Gadd45 is defective in ataxia telangiectasia. *Cell* **71**: 587–597

Kerr JFR, Winterford CM and Harmon BV (1994) Apoptosis. Its significance in cancer and cancer therapy. *Cancer* **73**: 2013–2026

Kuerbitz SJ, Plunkett BS, Walsh WV and Kastan MB (1992) Wild-type p53 is a cell cycle checkpoint determinant following irradiation. *Proc Natl Acad Sci USA* **89**: 7491–7495

Lee JM and Bernstein A (1993) p53 mutations increase resistance to ionizing radiation. *Proc Natl Acad Sci USA* **90**: 5742–5746

Levine AJ, Momand J and Finlay CA (1991) The p53 tumor suppressor gene. *Nature* **351**: 453–456

Lowe SW, Ruley HE, Jacks T and Housman DE (1993) p53-dependent apoptosis modulates the cytotoxicity of anticancer agents. *Cell* **74**: 957–967

Lowe SW, Schmitt EM, Smith SW, Osborne BA and Jacks T (1994) p53 is required for radiation-induced apoptosis in mouse thymocytes. *Nature* **362**: 847–849

Merritt AJ, Potten CS, Kemp CJ, Hickman JA, Balmain A, Lane DP and Hall PA (1994) The role of p53 in spontaneous and radiation-induced apoptosis in the gastrointestinal tract of normal and p53-deficient mice. *Cancer Res* **54**: 614–617

Milas L, Stephens LC and Meyn RE (1994) Relation of apoptosis to cancer therapy. *In Vivo* **8**: 665–673

Miyashita T and Reed CR (1995) Tumor suppressor p53 is a direct transcriptional activator of the human bax gene. *Cell* **80**: 293–299

Momand J, Zambetti GP, Olson DC, George D and Levine AJ (1992) The mdm-2 oncogene product forms a complex with the p53 protein and inhibits p53-mediated transactivation. *Cell* **69**: 1237–1245

Neto Jar, Quilici FA and Rois JA (1989) A comparison of non-operative versus preoperative radiotherapy in rectal carcinoma: a 10-year randomised trial. *Dis Colon Rectum* **32**: 702–710

Nevaldine B, Longo JA and Hahn PJ (1994) Hyperthermia inhibits the repair of DNA double-strand breaks induced by ionizing radiation as determined by pulsed-field gel electrophoresis. *Int J Hyperthermia* **10**: 381–388

Pahlman L and Glimelius B (1990) Pre- or postoperative radiotherapy in rectal and rectosigmoid carcinoma. *Ann Surg* **211**: 187–195

Shaw P, Bovey R, Tardy S, Sahli R, Sordat B and Costa J (1992) Induction of apoptosis by wild-type p53 in a human colon tumor derived cell line. *Proc Natl Acad Sci USA* **89**: 4495–4499

Shchpotin IB, Soldatenkov V, Buras RR, Nauta RJ, Shabahang M and Evans SRT (1994) Apoptosis of human primary and metastatic colon adenocarcinoma cell lines in vitro induced by 5-fluorouracil verapamil, and hyperthermia. *Anticancer Res* **14**: 1027–1032

Slichenmeyer WJ, Nelson WG, Slebos RJ and Kastan MB (1993) Loss of a p53-associated G1 checkpoint does not decrease cell survival following DNA damage. *Cancer Res* **53**: 4164–4168

Stockholm Rectal Cancer Study Group (1990) Preoperative short-term radiation therapy in operable rectal carcinoma: a preoperative randomised trial. *Cancer* **66**: 49–55

Takano YS, Harmon BV and Kerr JFR (1991) Apoptosis induced by mild hyperthermia in human and murine tumour cell lines: a study using electron microscopy and gel electrophoresis. *J Pathol* **163**: 329–336

© Cancer Research Campaign 1998

Tomasovic SP, Vasey TA, Story MD, Stephens LC and Klostergaard J (1994) Cytotoxic manifestations of the interaction between hyperthermia and TNF: DNA fragmentation. *Int J Hyperthermia* **10**: 247–262

Takahashi T, Kohno K, Yamaguchi T and Narisawa T (1982) Preoperative irradiation and 5-fluorouracil suppository for carcinoma of the rectum. *Am J Surg* **143**: 183–185

Takahashi T, Horie H, Kojima O and Itoh M (1993) Preoperative combined treatment with radiation, intraluminal hyperthermia, and 5-fluorouracil suppositories for patients with rectal cancers. *Surg Today* **23**: 1043–1048

Wijsman JH, Jonker RR, Keijzer R, van de Velde CJ Cornelisse CJ and van Dierendonck JH (1993) A new method to detect apoptosis in paraffin sections: in situ end-labeling of fragmented DNA. *J Histochem Cytochem* **41**: 7–12

*© Cancer Research Campaign 1998*